# Association among Orthodontic Malocclusions, Paranasal Sinuses Anatomic Variations and Adenoid Vegetation in Children Using CBCT

**DOI:** 10.3390/children10091549

**Published:** 2023-09-14

**Authors:** Serdal Kenan Kose, Secil Aksoy, Merve Onder, Ulas Oz, Kaan Orhan

**Affiliations:** 1 Faculty of Medicine, Department of Biostatistics, Ankara University, Ankara 06600, Türkiye; 2 Faculty of Dentistry, Department of Dentomaxillofacial Radiology, Near East University, 99138 Nicosia, North Cyprus; 3 Faculty of Dentistry, Department of Dentomaxillofacial Radiology, Ankara University, Ankara 06500, Türkiye; merveonder_16@hotmail.com (M.O.);; 4 Faculty of Dentistry, Department of Orthodontics, International Final University, 99138 Nicosia, North Cyprus

**Keywords:** adenoid vegetation, CBCT, children, orthodontic malocclusion, paranasal sinus anatomic variations

## Abstract

The aim of this study is to evaluate the relationship between orthodontic malocclusion, paranasal sinus (PS) variations, and adenoid vegetation in a group of pediatric patients with chronic rhinosinusitis. Clinical and radiographical data were retrospectively evaluated and 58 patients were diagnosed as having chronic sinus disease. Cone-beam computed tomography (CBCT) images were acquired with Newtom-3G. Anatomical variations of the PS were assessed on every section. Additionally, for cephalometric analysis, the images were imported into the InVivoDental software program. A total of 252 anatomical variations, which encompassed 19 different types, were detected in the current study. Concha bullosa was the most common anatomical variation, at 72.4%. Septum deviation was the second most common one, at 67.2%. The Class III group exhibited a significantly higher prevalence of concha bullosa and secondary middle turbinate than the other groups. While adenoid vegetation was most common in the Class III group, sinusitis and antral disease were most common in the Class II group. Overall, Class III subjects exhibited fewer PS variations. In conclusion, concha bullosa emerged as the most prevalent anatomical variation, with distinctive patterns observed across different malocclusion groups. Therefore, CBCT is useful, especially in pediatric patients, due to its low dose advantage.

## 1. Introduction

Embryologically, paranasal sinuses arise from the cartilaginous nasal capsule by budding in the early weeks of gestation, however major pneumatization occurs after birth [1]. The maxillary sinus is the first paranasal sinus to develop. Its development begins in the third week of gestation (around 13th to 14th weeks) [1]. The primordial maxillary sinus arises from a channel near the primordial ethmoid infundibulum on the lateral side of the uncinate process within the middle meatus [2].

The development of the primordial ethmoidal bulla occurs during the 12th week of gestation [3]. This bulla manifests as a cartilaginous projection located on the lateral wall of the middle meatus [1,2,3]. This area serves as the primary location for the formation of anterior ethmoidal cells, yet the posterior ethmoidal cells arise from the posterior aspect of the ethmoidal infundibulum between 16 and 20 weeks gestation [3]. The maxillary and ethmoid sinuses are observed to be present at birth [4]. However, it is noteworthy that the development of the sphenoid sinus starts shortly after birth. It is formed by the posterior ethmoidal cells [3,4]. The frontal sinus is the final paranasal sinus to undergo development, typically occurring at approximately 5 years of age [3].

Paranasal sinuses grow along with the nasal cavity and other facial structures and reach their mature size during adolescence [5,6]. During this period, paranasal sinuses play a critical role in various physiological functions, including voice resonance, thermal regulation of inhaled air, and reducing cranial weight [7,8]. As the facial structures evolve during childhood and adolescence, any variations in the development of the sinuses may have implications for overall health and facial skeletal growth.

Adenoid tissue plays a vital role in enhancing mucosal immunity by producing immunoglobulins. Nevertheless, in cases of adenoid hypertrophy, it can lead to nasal obstruction and serve as a reservoir for infectious microorganisms. As a result, adenoid tissue is commonly acknowledged as a significant factor in the development of childhood infections, including sinusitis, pharyngitis, and otitis media [9].

The presence of chronic sinusitis in pediatric patients has a significant impact on their overall quality of life, leading to a substantial burden. Various perspectives exist on the underlying pathogenesis of this condition, leading to continued debate over its etiology. Sinusitis is characterized by the presence of inflammation-induced swelling of the mucosal lining, obstruction of the sinus drainage pathway, and impaired functioning of the mucociliary clearance mechanism. A notable association has been observed between this particular condition and recurrent upper respiratory infections, alongside a range of factors including allergic and nonallergic rhinitis, cystic fibrosis, immunodeficiency, and anatomical abnormalities [10].

Various pathologies, such as the existence of Haller cells, agger nasi cells, and concha bullosa, have been identified as potential factors that could narrow the drainage pathway, potentially increasing the susceptibility of patients to chronic sinusitis [11]. Several studies reviewed the association between anatomic variations and rhinosinusitis in children; though a certain connection between them was not established. Various factors can be related to rhinosinusistis, such as infections, allergies, anatomic abnormalities, immune deficiencies, and mucociliary transport disorders [1,11,12]. Anatomical variations, although playing a minor role, should also be considered in the context of pediatric sinusitis [12,13].

Anatomical variations of the paranasal sinuses, such as concha bullosa and nasal septal deviation, may impair airflow and contribute to the development of rhinosinusitis in both the children and adults [10,14]. In growing children, altered facial skeletal development may be more prone to such variations [15,16,17].

Adenoid hypertrophy can cause nasal congestion, impairing nasal airflow and mucociliary clearance. This may predispose to rhinosinusitis and may also affect facial skeletal development and malocclusion [1,9]. Chronic rhinosinusitis itself can adversely affect craniofacial growth and development, potentially leading to malocclusions.

Different imaging modalities, including computed tomography (CT) and cone-beam computed tomography (CBCT), are used to evaluate paranasal sinus anatomy, pathology, and variations. While CT is considered the gold standard, CBCT presents certain advantages including lower radiation exposure, isotropic voxels, decreased metallic artifacts, and cost-effectiveness [18].

In the literature, there is limited research evaluating the correlation between paranasal sinus anatomic variations and orthodontic malocclusions [19]. Moreover, we could not find any studies specifically investigating anatomic variations and orthodontic malocclusions in rhinosinusitis patients using cone-beam CT. Hence, the null hypothesis of this study was that there were no significant differences in paranasal sinus anatomic variations and adenoid vegetation between the orthodontic malocclusion groups.

## 2. Materials and Methods

The study protocol was conducted in accordance with the principles outlined in the Declaration of Helsinki, including all relevant amendments and revisions. Access to the collected data was restricted to the investigators only. The study protocol was reviewed and approved by the Health Sciences Ethics Committee of the Near East University (IRB No: YDU/2022/109-1679). We retrospectively evaluated the clinical and radiographic data of 58 patients who were admitted to pediatrics and otorhinolaryngology departments of Faculty Hospital from 2009–2011. The anamnesis and clinical data were obtained from the corresponding department archive whereas the radiographic data were obtained from the archive data of the Dentomaxillofacial Radiology Department.

Using retrospective data from our faculty, a power analysis (Power and Precision software, Biostat, Englewood, NJ, USA) was conducted that indicated that detection of differences between the malocclusions and anatomical landmarks could be obtained with 50 patients at a power of 0.8 (α = 0.05). Thus, this study was conducted using 58 selected good-quality CBCT images.

A total of 30 female and 28 male patients, aged between 9 and 16, with a mean age of 14.03 ± 2.44 years, were diagnosed with chronic sinus disease.

Inclusion criteria for patients were as follows:

- Patient under 17 years of age;
- Presence of the clinical diagnosis of chronic sinus disease;
- Presence of persistent sinonasal symptoms lasting for more than three months, such as nasal discharge, headache, cough, or nasal obstruction.

This evaluation was conducted by the relevant otorhinolaryngology department.

Exclusion criteria for patients were as follows:

- Patient had a prior medical history of surgery or trauma;
- Antrochoanal polyps, cystic fibrosis, asthma, or immune deficiency;
- Any tumor, or malignancy in the head and neck region.

The CBCT images were acquired using a Newtom-3G CBCT machine manufactured by Quantitative Radiology s.r.l. in Verona, Italy (Parameters: tube voltage 120 kVp, tube current 3–5 mA, scan time 16.4 s, field of view (FOV) 9 or 12 inches, and axial slice thickness 0.4 mm). The CBCT scans were conducted in adherence to a rigorous standardized scanning protocol, with the patient positioned horizontally and instructed to keep their mouth closed and remain still throughout the duration of the scans.

Images were reconstructed using a 21.3-inch flat-panel color active matrix TFT medical monitor (Nio Color 3MP, Barco, Nanterre, France), featuring a resolution of 76 Hz and a pixel pitch of 0.2115 mm, operating at a 10-bit depth. Axial, coronal and sagittal sections were used to determine the presence of the paranasal sinus variations listed in Table 1 using the machine’s software (NNT viewer 4.2, Verona, Italy). Reconstructed sagittal CBCT images were used to evaluate the adenoid hypertrophy.

The CBCT scans were collected and evaluated using the Lund–Mackay staging system. The Lund–Mackay staging system assigns scores to each sinus, including the anterior ethmoid, posterior ethmoid, maxillary, frontal, and sphenoid sinuses. These scores are based on a scale that ranges from 0 (indicating no opacification) to 2 (indicating complete opacification). The scans were assessed by two maxillofacial radiologists (KO, SA) to identify any anatomical variations on both sides separately and then a consensus session was made in order to finalize the anatomical variations and Lund–Mackay scores. Moreover, before starting the radiographic examination in the study, the examiners were calibrated to recognize, as well as to identify, the anatomical variations. For such purpose, a 20 different CBCT scans, in addition to those in this study, were used. The examiners only examined the CBCTs and were blinded to any other patient data in the radiographic examination procedure. Furthermore, in order to conduct cephalometric analysis, the images obtained from the Newtom 3G dataset were exported in the DICOM file format and subsequently imported into the InVivoDental (Anatomage®, San Jose, CA, USA) software program. All cephalometric measurements were done by two dentomaxillofacial radiologists with 20 and 10 years of experience (K.O., S.A.).

Moreover, the utilization of software facilitated the creation of a novel template designed specifically for conducting a 3D cephalometric analysis, which was subsequently employed for evaluating the cephalometric characteristics of the subjects. The classification of malocclusion into Class I, Class II, and Class III is based on the measurement of the ANB angle. Class I malocclusion is characterized by an ANB angle ranging from 0° to 4°. Class II malocclusion is identified by an ANB angle greater than 4°, whereas Class III malocclusion is characterized by an ANB angle less than 0°.

### 2.1. Statistical Analyses

All anatomic variations, rhinosinusitis, adenoids, and malocclusion classes were reported by frequency (%). The associations between malocclusion and anatomic variations, rhinosinusitis, and adenoids were assessed by Fisher–Freeman–Halton test. A *p*-value < 0.05 was considered as statistically significant. The analyses were conducted using IBM SPSS Statistics 22.0 software (IBM Corp., 2013, Armonk, NY, USA).

### 2.2. Examiner Reliability and Statistical Analysis

Intra- and inter-examiner validation for cephalometric measurements were conducted. To assess intra-observer reliability, the Wilcoxon matched-pairs signed-rank test was used for repeat measurements. The inter-observer reliability was determined by the intraclass correlation coefficient (ICC) and the coefficient of variation (CV) [CV = (standard deviation/mean) × 100%]. Values for the ICC range from 0 to 1. ICC values greater than 0.75 show good reliability, and the low CV demonstrates the precision error as an indicator of reproducibility [20].

## 3. Results

### 3.1. Intra-Observer Consistency

Repeated CBCT evaluation and measurements indicated no significant intra-observer difference for both observers (*p* > 0.05). Overall intra-observer consistency for observer 1 was rated at 92% and 94%, while the consistency for observer 2 was found 90% and 92% between the two evaluations and measurements, respectively. All measurements were found to be highly reproducible for both observers and no significant difference was obtained from two measurements of the observers (*p* > 0.05).

### 3.2. Inter-Observer Consistency

The ICCs between Observer 1 and Observer 2 ranged from 0.90 to 0.92. There was high inter-observer agreement, while a high ICC and low CV demonstrated that the procedure was standardized between the evaluations and measurements of the observers. No statistical differences were found among observers’ evaluations and measurements (*p* < 0.05). Observer 1 had the highest intra-observer consistency, thus the mean of this observer’s evaluations and measurements were chosen for further analysis.

**Figure 1:**
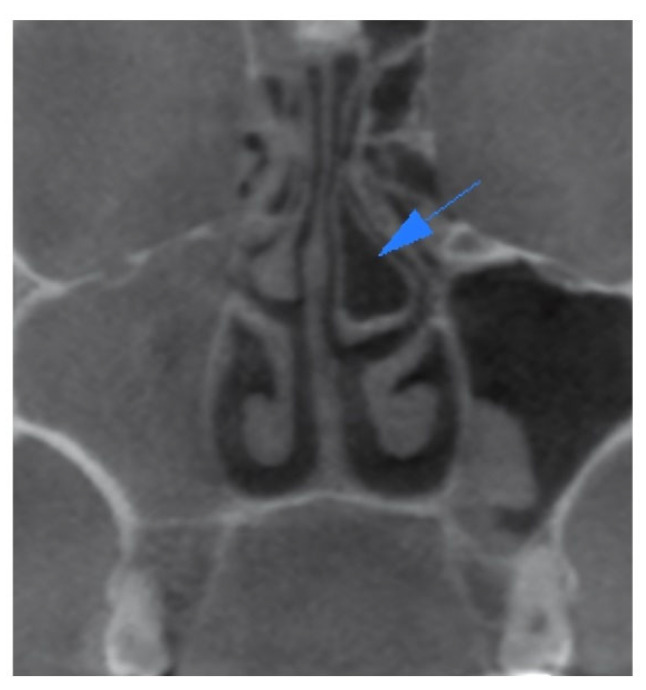
Coronal CBCT scan showing the pneumatization of the middle turbinate, which can be classified as bulbous type of concha bullosa, on the left side marked with blue arrow.

**Figure 2:**
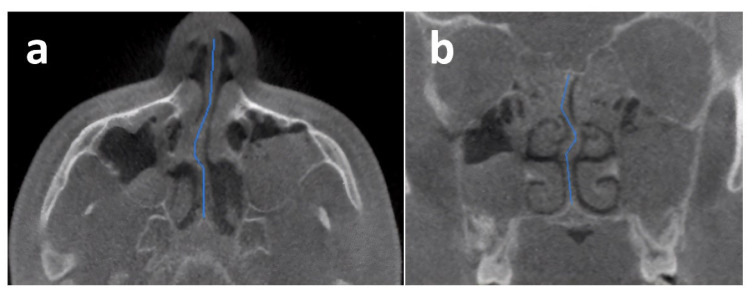
Axial (a) and coronal (b) CBCT scan showing the nasal septum deviation drawn with blue line.

**Figure 3:**
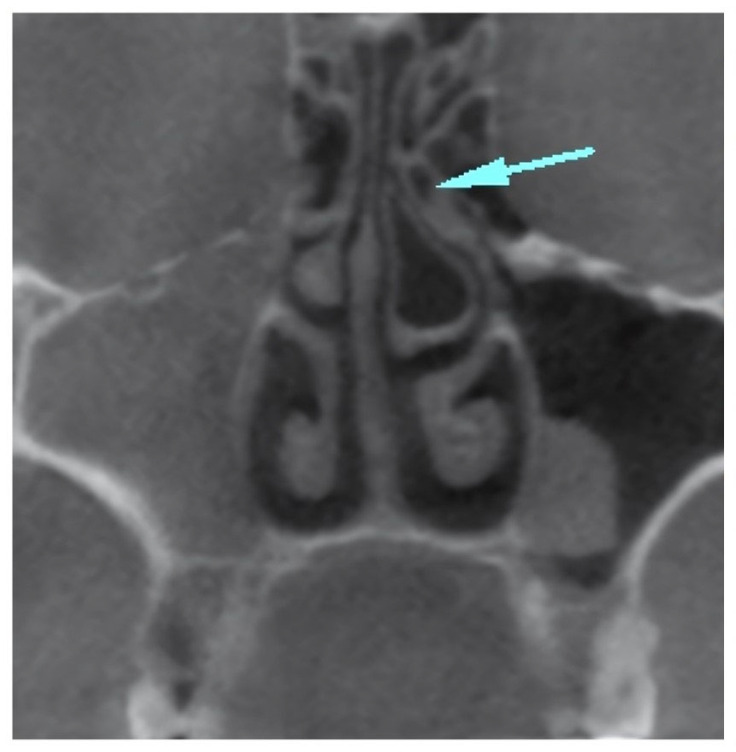
Blue arrow marking the pneumatization of the uncinate process, which is also called uncinated bulla, on the left side.

In the present study, a comprehensive analysis revealed the identification of a total of 252 anatomical variations, which encompassed nineteen different types. The most prevalent anatomical variation observed in the current study was concha bullosa, accounting for 72.4% of cases (Figure 1). The second most prevalent condition observed was septum deviation, accounting for 67.2% of cases (Figure 2). This was followed by uncinate bulla (Figure 3), nasal septum pneumatization, superior concha bullosa, and Haller cell.

Table 1 presents a comprehensive summary of the frequency of these anatomical variations observed among the participants in the study. Statistically significant differences were observed in the prevalence of concha bullosa and secondary middle turbinate among different orthodontic malocclusion groups. The Class III group exhibited a significantly higher prevalence of concha bullosa and secondary middle turbinate than the other malocclusion groups. On the other hand, the presence of concha bullosa was significantly lower in the Class II group compared to the other groups. Overall, Class III subjects exhibited fewer paranasal sinus variations than the other malocclusion groups, although the difference was not statistically significant.

The statistical analysis did not reveal a significant correlation between the different skeletal malocclusions and the presence of adenoid vegetation despite the higher occurrence of adenoid vegetation in the Class III malocclusion group. The Class II group exhibited the highest prevalence of sinusitis and antral disease, followed by the Class III and Class I groups, as indicated in Table 2.

## 4. Discussion

In the current study, numerous anatomical variations in the paranasal region were observed. Concha bullosa was the most prevalent, followed by septum deviation and uncinate bulla. Furthermore, different orthodontic malocclusions presented distinct anatomical variations. For instance, the Class II group exhibited a lower presence of concha bullosa compared to other malocclusion groups. Conversely, the Class III group had a higher prevalence of both concha bullosa and secondary middle turbinate than other malocclusion groups. The observed variations in paranasal sinus not only have clinical significance in terms of orthodontic malocclusions but also bear relevance to respiratory patterns. The presence or absence of certain anatomical variations, such as concha bullosa or secondary middle turbinate, could potentially influence airflow dynamics in the nasal passages. It is essential to consider how these anatomical differences could potentially influence other aspects of health, such as breathing patterns and the development of malocclusions. This broader context brings attention to studies that have delved into the relationship between nasal airflow, adenoid tissue size, and malocclusions. For example, Linder- Aranson’s respiratory mode study series revealed that there is a negative association between nasal airflow and adenoid tissue size. The series focused on adenoid size and its relationship with oral and nasal breathing type. They concluded that, after surgical removal of adenoid tissue, the majority of subjects changed breathing type from mouth to nose [15]. Interestingly, the literature declares that malocclusion by itself may also contribute to reducing nasal airflow. The association between breathing patterns and the development of malocclusion was emphasized by Occasi et al. [14]. Hence, the development of the facial structure and its correlation with the type of breathing, whether oral or nasal, is typically influenced by variations in the anatomy of the paranasal sinuses, chronic rhinosinusitis, and the presence of adenoid vegetation [14,15].

It has been suggested that anatomical variations present in the nasal cavity and paranasal sinus may have the potential to contribute to the obstruction of the ostiomeatal unit. In order to investigate this hypothesis, a research study conducted by Kim et al. utilized computed tomography (CT) scans to assess the scope and pattern of sinus disease, along with any accompanying anatomical variations, in a group of 113 children [11]. The findings of this study revealed a lack of statistically significant association between sinusitis and anatomical variations. Similarly, Alkire et al. conducted a comprehensive investigation of 78 temporal CT scans obtained from pediatric patients with sinusitis and discovered that the existence of Haller cells and narrower infundibular widths were significantly related to recurrent rhinosinusitis when compared to control subjects [13]. Furthermore, Sivaslı et al. have documented that, although there was no statistically significant association between sinusitis and anatomical variations, they did observe a positive correlation between maxillary sinusitis and an enlarged ethmoidal bulla [1].

The presence of an air-filled cavity in the superior, middle, and inferior turbinate is known as superior concha bullosa, concha bullosa, and inferior concha bullosa, respectively. The classification of pneumatization of the middle turbinate is based on the location of aeration and can be categorized into three groups: lamellar, bulbous, and extensive, as described in certain studies [21,22]. Although there is a possibility for these anatomical variations to result in the obstruction of the normal airway passage in the middle nasal meatus and recurrent ethmoiditis, no correlation has been identified between them [23].

These anatomical variations, which disrupt nasal drainage, make breathing difficult, and sometimes cause skeletal disorders, may require surgical intervention in cases of severe discomfort. Turbinate surgery, which is one of these surgeries and also known as turbinate reduction, is a procedure that aims to relieve nasal blockage by shrinking the size of the inferior, middle, and/or superior nasal turbinates. It may be performed as a standalone procedure or in conjunction with sinus surgery. In a study conducted by Cocuzza et al., it was seen that this surgery gave good results that were similar to other sinus surgeries [24].

Previous studies conducted on pediatric populations have provided estimates for the prevalence of middle concha pneumatization ranging from 39.4% to 58% [1,11,25]. Furthermore, the results of the current study indicate that the most prevalent anatomical variation is concha bullosa, and all subjects in the Class III malocclusion group display this anatomic variation. Nasal septum deviation ranks as the second most prevalent condition, discovered in 67.2 percent of cases. Several researchers have investigated the correlation between septum deviation, concha bullosa, and/or paranasal sinusitis [26,27]. Stallman and colleagues have observed a significant association between the presence of a unilateral or dominant concha and contralateral septum deviation. However, they did not establish any significant relation between concha bullosa and paranasal sinus disease [26]. Furthermore, Smith et al. conducted a study examining 883 CBCT scans to determine the prevalence of concha bullosa and nasal septum deviation, as well as their potential association with maxillary sinusitis. However, their findings did not indicate any statistically significant association between these conditions [27].

Therefore, whether skeletal hard tissue or respiratory airflow originates from soft tissue, they are affected by each other and create a pathogenic process in the craniofacial system [28]. In the current study, we tried to examine the effect of anatomic variation in rhinosinusitis, regardless of skeletal structure. Considering that concha bullosa is an air-filled swelling of hard tissue surrounded by nasal passage soft tissues, a greater functional component of activity might be expected. In their study, Park et al. discussed the ‘e vacuo theory’ proposed by Stammberger, which suggests a relationship between airflow reduction in the nasal cavity due to nasal septum deviation and the pneumatization of the contralateral nasal concha [19]. Hence, the presence of an obstruction in the airflow can result in a persistent habit of breathing through the mouth, consequently leading to alterations in the positioning of the head and jaw in a downward and backward direction [19]. Such positional changes of craniofacial soft tissues and functional components can affect the development of craniofacial hard tissues [19]. This theory is also supported strongly by Moss and Salentijn [16]. Hence, functional components can exert an influence on the adaptive responses observed in the development of craniofacial skeletal structures. However, upon further examination of Stamberger’s second theory, it becomes apparent that anatomical variations, such as nasal septum deviation and concha bullosa, are not interrelated and do not have a causal relationship with one another [19]. If we consider these two different approaches, there is a possible association between anatomic variations (e.g., nasal septum deviation, and/or concha bullosa) and supporting hard tissue pneumatization.

Hence, from a clinical standpoint, it is advisable to monitor anatomical variations, particularly in Class III patients, and incorporate a thorough evaluation and potential treatment for Class III skeletal malocclusions to improve treatment effectiveness. These considerations are particularly crucial for ensuring post-treatment stability.

This study has certain limitations that ought to be considered when interpreting its findings. First, the sample size is relatively small, restricting the scope and potential generalizability of the results. The implications and associations identified here would be more persuasively elucidated if substantiated with a larger population. Moreover, this study lacks a control group comprising patients without sinusitis, an omission that might compromise the contrast and comparison of the findings. For future research, it would be beneficial to incorporate such a control group to enable more definitive and broadly applicable conclusions. These limitations, while notable, serve as guiding points for subsequent investigations, underlining the need for broader and more comprehensive studies in this domain.

## 5. Conclusions

In conclusion, the null hypothesis of the current study was not completely rejected as significant differences were found for concha bullosa and secondary middle turbinate between the malocclusion groups. Various anatomical variations within the paranasal region and their potential implications for orthodontic malocclusions were assessed. Notably, concha bullosa emerged as the most prevalent anatomical variation, with distinctive patterns observed across different malocclusion groups. CBCT emerges as a potent radiographic method for the correct identification of anatomical variations. Notably, if necessary, its strength lies in providing precise imaging while minimizing ionizing radiation exposure, rendering it particularly advantageous for children and contributing to enhanced clinical decision-making.

## Figures and Tables

**Table 1:** Distribution of paranasal sinus anatomic variations among orthodontic malocclusions.

	Malocclusion Class				
Anatomic	I	II	III	Total	
Variations	*n* (%)	*n* (%)	*n* (%)	*n* (%)	*p*-Value
Onodi cell					0.204
Absent	23 (76.7)	16 (88.9)	10 (100)	49 (84.5)	
Present	7 (23.3)	2 (11.1)	0 (0.0)	9 (15.5)	
Haller cell					0.423
Absent	22 (73.3)	10 (55.6)	7 (70)	39 (67.2)	
Present	8 (26.7)	8 (44.4)	3 (30)	19 (32.8)	
Agger nasi cell					0.421
Absent	20 (66.7)	13 (72.2)	9 (90)	42 (72.4)	
Present	10 (33.3)	5 (27.8)	1 (10)	16 (27.6)	
Interfrontal sinus septa cells					0.861
Absent	27 (90)	15 (83.3)	9 (90)	51 (87.9)	
Present	3 (10)	3 (16.7)	1 (10)	7 (12.1)	
Supraorbital ethmoid					0.540
Absent	27 (90)	14 (77.8)	8 (80)	49 (84.5)	
Present	3 (10)	4 (22.2)	2 (20)	9 (15.5)	
Superior concha bullosa					0.524
Absent	18 (60)	13 (72.2)	8 (80)	39 (67.2)	
Present	12 (40)	5 (27.8)	2 (20)	19 (32.8)	
Concha bullosa					0.003
Absent	6 (20)	10 (55.6) ^1^	0 (0.0)	16 (27.6)	
Present	24 (80)	8 (44.4)	10 (100) ^1^	42 (72.4)	
Secondary middle turbinate					0.030
Absent	30 (100)	17 (94.4)	8 (80)	55 (94.8)	
Present	0 (0.0)	1 (5.6)	2 (20) ^1^	3 (5.2)	
Bifid middle turbinate					NA
Absent	30 (100)	18 (100)	10 (100)	58 (100.0)	
Present	0 (0.0)	0 (0.0)	0 (0.0)	0 (0.0)	
Inferior concha bullosa					0.150
Absent	30 (100)	16 (88.9)	9 (90)	55 (94.8)	
Present	0 (0.0)	2 (11.1)	1 (10)	3 (5.2)	
Ethmomaxillary sinus					0.571
Absent	29 (96.7)	16 (88.9)	10 (100)	55 (94.8)	
Present	1 (3.3)	2 (11.1)	0 (0.0)	3 (5.2)	
Uncinate bulla					0.677
Absent	17 (56.7)	9 (50)	7 (70)	33 (56.9)	
Present	13 (43.3)	9 (50)	3 (30)	25 (43.1)	
Spheno-maxillary plate					0.254
Absent	27 (90)	14 (77.8)	10 (100)	51 (87.9)	
Present	3 (10)	4 (22.2)	0 (0.0)	7 (12.1)	
Maxillary sinus hypoplasia					0.422
Absent	28 (93.3)	18 (100)	9 (90)	55 (94.8)	
Present	2 (6.7)	0 (0.0)	1 (10)	3 (5.2)	
Frontal sinus hyperplasia					0.999
Absent	27 (90)	17 (94.4)	9 (90)	53 (91.4)	
Present	3 (10)	1 (5.6)	1 (10)	5 (8.6)	
Frontal sinus hypoplasia					0.282
Absent	27 (90)	18 (100)	10 (100)	55 (94.8)	
Present	3 (10)	0 (0.0)	0 (0.0)	3 (5.2)	
Sphenoid sinus ICA bulging					0.600
Absent	21 (70)	11 (61.1)	8 (80)	40 (69.0)	
Present	9 (30)	7 (38.9)	2 (20)	18 (31.0)	
Nasal septum deviation					0.811
Absent	9 (30)	7 (38.9)	3 (30)	19 (32.8)	
Present	21 (70)	11 (61.1)	7 (70)	39 (67.2)	
Nasal septum pneumatization					0.544
Absent	17 (56.7)	13 (72.2)	7 (70)	37 (63.8)	
Present	13 (43.3)	5 (27.8)	3 (30)	21 (36.2)	
Crista galli pneumatization					0.999
Absent	29 (96.7)	18 (100)	10 (100)	57 (98.3)	
Present	1 (3.3)	0 (0.0)	0 (0.0)	1 (1.7)	

^1^ Significantly more frequent malocclusion class compared to the other category of anatomic variation in the corresponding row. *p*-value less than 0.05 shows statistically significant differences.

**Table 2:** Distribution of adenoid vegetation and chronic rhinosinusitis among orthodontic malocclusions.

	Malocclusion Class				
	I	II	III	Total	
	*n* (%)	*n* (%)	*n* (%)	*n* (%)	*p*-Value
Sinusitis antral disease					0.199
Absent	18 (60.0)	6 (33.3)	4 (40.0)	28 (48.3)	
Present	12 (40.0)	12 (66.7)	6 (60.0)	30 (51.7)	
Adenoid					0.229
Absent	19 (63.3)	10 (55.6)	3 (30)	32 (55.2)	
Present	11 (36.7)	8 (44.4)	7 (70)	26 (44.8)	

*p*-value less than 0.05 shows statistically significant differences.

## Data Availability

The data presented in the present study are available on request from the corresponding author. The data are not publicly available due to ethical considerations.
